# Filling gaps in animal welfare assessment through metabolomics

**DOI:** 10.3389/fvets.2023.1129741

**Published:** 2023-02-28

**Authors:** Maria Pia Fabrile, Sergio Ghidini, Mauro Conter, Maria Olga Varrà, Adriana Ianieri, Emanuela Zanardi

**Affiliations:** ^1^Department of Food and Drug, University of Parma, Parma, Italy; ^2^Department of Veterinary Science, University of Parma, Parma, Italy

**Keywords:** Five Domains model, biomarkers, metabolomics workflow, NMR-based metabolomics, HRMS-based metabolomics, animal-based measures

## Abstract

Sustainability has become a central issue in Italian livestock systems driving food business operators to adopt high standards of production concerning animal husbandry conditions. Meat sector is largely involved in this ecological transition with the introduction of new label claims concerning the defense of animal welfare (AW). These new guarantees referred to AW provision require new tools for the purpose of authenticity and traceability to assure meat supply chain integrity. Over the years, European Union (EU) Regulations, national, and international initiatives proposed provisions and guidelines for assuring AW introducing requirements to be complied with and providing tools based on scoring systems for a proper animal *status* assessment. However, the comprehensive and objective assessment of the AW *status* remains challenging. In this regard, phenotypic insights at molecular level may be investigated by metabolomics, one of the most recent high-throughput omics techniques. Recent advances in analytical and bioinformatic technologies have led to the identification of relevant biomarkers involved in complex clinical phenotypes of diverse biological systems suggesting that metabolomics is a key tool for biomarker discovery. In the present review, the Five Domains model has been employed as a *vademecum* describing AW. Starting from the individual Domains—nutrition (I), environment (II), health (III), behavior (IV), and mental state (V)—applications and advances of metabolomics related to AW setting aimed at investigating phenotypic outcomes on molecular scale and elucidating the biological routes most perturbed from external solicitations, are reviewed. Strengths and weaknesses of the current state-of-art are highlighted, and new frontiers to be explored for AW assessment throughout the metabolomics approach are argued. Moreover, a detailed description of metabolomics workflow is provided to understand dos and don'ts at experimental level to pursue effective results. Combining the demand for new assessment tools and meat market trends, a new cross-strategy is proposed as the promising combo for the future of AW assessment.

## 1. Introduction

In recent years, animal welfare (AW), primarily related to food-producing animals, became a relevant topic of public health due to the overall impact on the condition of the animals, with consequences for disease, productivity, and food safety (1). The 13th article of the Treaty on the Functioning of the European Union (EU) marked a new beginning in the EU policy concerning AW, recognizing animals as sentient beings (2). Over the years, the debate on this issue has involved several international organizations that have focused their attention on awareness themes aiming to promote a state of wellbeing affecting human, animals, and environment. In this context, it is worth mentioning *2030 Agenda for Sustainable Development* adopted in 2015 by the members of the United Nations, a 15-years plan of action for people, planet, and prosperity expected to guide the actions of the international community. Among its seventeen sustainable development goals, some are related to AW and food consumption, such as goal #3 “Good health and well-being,” goal #12 “Responsible consumption and production,” and goal #15 “Life on land” (3). This framework fits into the concept of One Health, the multisectoral and multidisciplinary approach adopted by the tripartite collaboration between the Food and Agriculture Organization of the United Nations, the World Organisation for Animal Health (WOAH), and the World Health Organization to address health threats at the human-animal-environment interface facing the world today (4). In this context, a central role is played by the international standards set by WOAH related to different aspects of AW for disease control purposes (5). The WOAH science-based standards embrace manifold issues of AW that at European level have been regulated to encourage measures for the protection of food producing animals kept for farming purposes (6), during transport and related operations (7), and at the time of killing (8). However, despite numerous international initiatives aiming to promote and achieve a welfare *status*, one of the main challenges remains uniquely identifying a welfare's condition; this limitation is due to the multidisciplinary nature of AW assessment, given that it concerns aspects related to behavioral and cognitive sciences, animal husbandry, veterinary pathology, biochemistry, physiology, and nutrition (9).

In the EU, claims related to animal husbandry conditions, included those related to AW, are voluntary indications more and more often indicated on meat labels to meet consumer's demand for a transition toward more ethical and sustainable farming systems. European consumers' initiatives, such as that entitled “End of the Cage Age” (10) evidence the importance of ethical values related to AW issue defining the so-called *untouchable quality* to be complied with for the food market. Communication about AW is a current requirement for food business operators encouraged by this increased awareness. A study on AW labeling for the European Commission showed that at least half of the European consumer population would like to receive information on the conditions under which farmed animals are kept and treated. Currently, fifty-one labels with AW claims present in the EU Member States and the UK were monitored (11). In the case of beef, for labels containing indications other than mandatory, each operator or organization shall send a specification for approval to the competent authority of the Member State in which production or sale take place (12). In the case of other farm animal species, some national and international transversal standards encompass only a few aspects of AW marking the unavailability of harmonized approaches. All the standards, that may be voluntarily adopted by the operators, are usually represented by requirements to be met in order to pursue AW (13). Compliance with the management-based requirements (e.g., stock density, bedding type, water facilities) is evaluated using outcomes of scoring systems during periodic control visits. Moreover, animal-based measures are often included in the protocols and guidelines proposed to be implemented on farm and at slaughter to assess and improve welfare (14). Protocols are constantly evolving and improving; recently, the panel on Animal Health and Welfare of the European Food Safety Authority has suggested a set of animal-based measures to use at slaughter for monitoring on-farm welfare of cull sows and rearing pigs (15). In Italy, some protocols for AW assessment of various animal species—based on scoring grids of general farm management, structures, equipment, major risks, and animal-based measures—have been developed within ClassyFarm, an integrated system aimed at categorizing livestock farms (16). Moreover, an initiative called Il Sistema di Qualità Nazionale per il Benessere Animale has been recently drawn up throughout a concerted action between the Ministry of Health and the Ministry of Agriculture Food and Forestry Politics with the aim to achieve production standards beyond the legal limits and not just for AW (17).

The development of tools for an objective assessment of the AW is a topic of great interest to the food sector for the purposes of authentication and traceability of food of animal origin, and to implement efficient control systems by food business operators, competent authorities, and certification bodies. To date, stress blood levels of cortisol, creatine kinase, and lactate are used as common biomarkers of animal stress; a recent proteomic study enabled to demonstrate the relationship between some meat quality defects associated with AW, and some structural-contractile skeletal proteins have been proposed as biomarkers of heat stress during the rearing and pre-slaughter management for beef meat, pork, and poultry (18).

However, as reported by Keeling et al., the “one perfect indicator”, reflecting an integrative measure of both negative and positive welfare and placing an individual on a scale from having very poor to very good welfare, is still the holy grail of welfare assessment (19).

Because AW phenotypic understanding may be based upon what it can be observed about both the temperament of the animal and the metabolic changes, new phenotypic insights at molecular level may be investigated by metabolomics, one of the most recent high-throughput omics techniques. Metabolomics is a comprehensive analysis of endogenous and exogenous low-molecular-weight (typically <1,500 Da) metabolites in biological systems. Metabolites intended as precursors, intermediates, and products of biochemical processes, provide a molecular snapshot of the complex interplay between genome and environment; for this reason, metabolomics has been defined as the link between genotypes and phenotypes (20, 21).

Over the last decade advances in analytical and bioinformatic technologies have led to the identification of relevant biomarkers involved in complex clinical phenotypes of diverse biological systems suggesting that metabolomics is a key tool for biomarker discovery. In this respect, being welfare *status* the result of internal cellular activities that may be triggered by external stimuli, the metabolomics approach may help to gain molecular fingerprints related to AW by revealing metabolites and in which way their levels may change as a result of external conditions (22).

Therefore, the present review is aimed at highlighting the link between AW and metabolomics beyond the state-of-the-art and exploring new frontiers for AW assessment. In particular, the recent applications and advances of metabolomics in the AW context are argued. The methodological aspects of the metabolomic workflow, as well as strengths and weaknesses, are presented. Challenges and outlooks of metabolomics as a new tool for a comprehensive AW assessment are also discussed in the final part of the review.

## 2. Animal welfare from Five Freedoms paradigm to Five Domains model

A milestone in the description of AW dates back to 1965 when the Brambell Report introduced for the first time the Five Freedoms paradigm, later considered also by the British farm animal welfare Council in 1979. That paradigm established five macro-areas of freedom to satisfy aiming to guarantee the animal a life worth living and free animals from discomforts (23). The Five Freedoms paradigm embodies several animal needs and establishes a view considering welfare just as a condition devoid of negative experiences without promoting positive experiences. This paradigm is based upon avoiding unfavorable conditions able to induce welfare-compromising negative effects. However, a good welfare *status* stands for reducing negative experiences and encouraging positive experiences along the entire life of the animal including, for example, feeds having attractive smells, access to preferred sites for resting, and protection during transport and at the slaughterhouse (24). Therefore, despite its acclaim, the Five Freedoms model carried a great limitation concerning a vision just limited on “what to avoid” and not “what to pursue” to guarantee AW; in keeping with this, the logic behind the paradigm was “animal should be free from thirsty” and not “provide the animal with adequate water supply”. Through the years, more and more attention has been paid to the provisions ensuring AW by leading to the development of the Five Domains model intended not as freedoms but in terms which highlighted welfare positive connotation (25). Through this latter model the description of AW passed from “animals free from thirst” to “good feedings” by introducing new ways and perspectives of approaching the issue (23, 26). The Five Domains model is accurately defined by “five Domains” that are, respectively, (I) nutrition, (II) environment, (III) health, (IV) behavior, and (V) mental state, seen as gear wheels which worked if wedged. The first three Domains mainly referred to physiological needs to be met from a nutritional, environmental, and health point of view. The vision changes with the fourth Domain—behavior—characterized by a faceted standpoint; in fact, if on the one hand, the behavior may be the answer to objective conditions, such as breeding type or environmental conditions, on the other hand, it could have a strong subjective connotation. The shift from objective to subjective connotation led to the fifth Domain—mental state—highly influenced both by the first four ones (24).

## 3. Metabolomics applications within animal welfare: Domain-by-Domain

**Figure 1:**
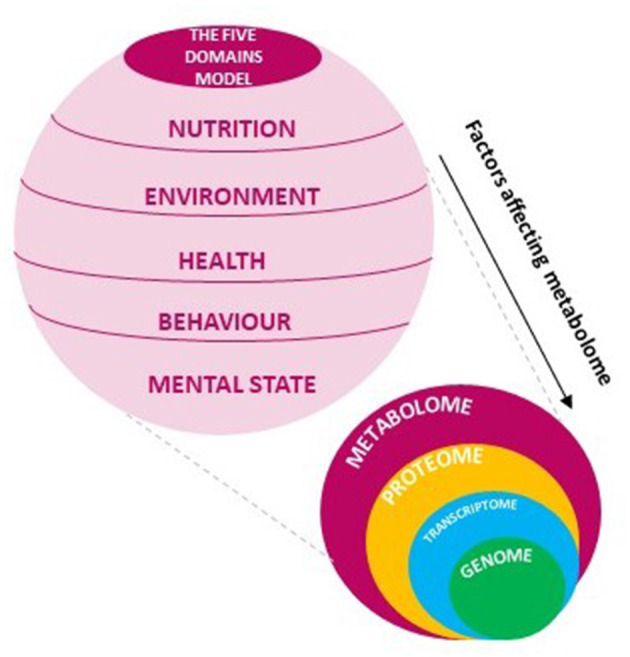
The Five Domains model embodies all factors affecting changes in metabolome that may be explored with -omics sciences.

Metabolomics is a young discipline grown in the last years within the omics techniques. Although many applications are already available in various scientific fields, this science has a great potential expected to be increasingly exploited for new scopes of research. Elucidating the structure of metabolites and relative interactions, metabolomics provides a global vision of a living organism strongly related to the phenotypic outcome (27). The phenotype understanding on molecular scale occurs by monitoring the changes in composition and levels of metabolites providing information on the cellular regulatory processes involved in the latest biological response of systems to genetic and environmental solicitation (28). Within the hierarchy of -omics approaches focused on the study of the molecular expressions of the biological systems, metabolomics is the most sensitive to variations, hence the best option to provide direct information about the physiological state of a living being. This high responsiveness allows to capture a snapshot of what happens at the metabolite level to discover biological pathways and related specific biomarkers, evidence of a well-defined condition (29, 30). For the scope of application, metabolomics in plant systems results widely explored demonstrating to be a valid tool when investigating the cause of biological effects, such as plant-pathogen interactions (20). In the same way, the effectiveness may suggest that metabolomics can be exploited to decipher the combination of “living organism-external stimuli” aiming to highlight the biological response to different conditions (Figure 1).

AW evaluation is based on a metrics considering a scale ranging from poor to good welfare, where the lower extreme is a condition in which the animal is suffering or stressed. With this in mind, AW is often investigated at the lower extreme, to clarify the biological mechanisms elicited when the animal perceives a threat to its homeostasis. In this regard, the time of exposure to the threat (short-term and long-term) is an important issue since living organisms cope with stress in different ways. Generally, the activity of sympathetic-adrenal medullary system, especially in the hypothalamic-pituitary-adrenal cortex system, is the focus for short-term stress studies, while enzyme activity and metabolites measurement for long-term stress (31). Usually, threats to the animal fall into one of the areas building the Five Domains model. However, free an animal from stress cannot be reduced to one Domain due to the very high interactivity of biological functions within the organism. Welfare Domains are strongly interrelated and therefore any form of disruptive instability concerning welfare has distinctive detectable using physiologically, pathologically, and clinically measurable outcomes (24). For instance, in an overcrowd pens, pigs will be most probably affected by heat stress (temperature, environment, Domain II), will struggle to grab food (nutrition, Domain I; agitation, mental state, Domain V), and cumulative stress will cause internal quarrels (behavior, Domain IV). Similarly, a loud noise in pen (auditory discomfort, environment, Domain II) will incite considerable fear in the animal (negative emotion, mental state, Domain V) that will assume an attitude of defense (behavior, Domain IV) since the noise is recognized by the animal as a threat (32). The last example clarifies the assumption of interactive Domains since an environmental stress (Domain II) induces a particular behavior (Domain IV) stimulated by different feelings (Domain V).

In line with this, some relevant examples from the scientific literature of metabolomics studies aimed at elucidating individually the role of factors belonging to defined Domains of the Five Domains model are listed in Table 1 and discussed in the following section. Although confined to single Domains, the study of metabolome allows to broaden the perspective, decipher comprehensive metabolic perturbations, leading ultimately to the identification of a specific biomarkers evidence of a general animal's *status*.

**Table 1:** Overview of metabolomics approach explored within the Five Domains model.

**Domain**	**Focus**	**Purpose of analysis**	**Species**	**Sample**	**AP**	**References**
	Feed supplementation	Study on changes in metabolites after dietary supplementation of arginine to basal diet in pigs reared in controlled facilities. Hypothesis to be tested: arginine provision may affect metabolites of AA, glucose and FA.	Pig	Serum	^1^H NMR	(33)
		Explore change in metabolic profile caused by the addition of sesamin in vegetable oils-based diets with varying *n*-6/*n*-3 FA ratio. Six different diets were compared for a period of 4-month feeding trial.	Salmon	Liver muscle	^1^H NMR	(34)
(I) Nutrition	Feed regimens	Characterize metabolome to ascertain pathways differences originating from grass-fed and grain-fed steers to understand possible implications in meat quality for public health and animal welfare.	Beef	Blood muscle	LC-MS GC-MS	(35)
	Feed deprivation	Assessment of metabolomic fingerprinting for malnutrition biomarkers identification in short-term fasted fish.	Sea bream	Serum	LC-MS	(36)
		Obtain a perspective of metabolites regulation during feed deprivation and understand metabolic fluxes characterizing fed and fasted fish, also monitoring growth and energy stores.	Trout	Plasma liver muscle	^1^H NMR	(37)
(II) Environment	Heat stress and RH	Determine a comprehensive impact of heat stress on lipid and non-lipid metabolites to identify signaling molecules of adaptation to heat stress.	Pig	Adipose tissue	LC-MS	(38)
			Study to identify metabolic pathways associated with the hot-humid or dry climate.	Chick	Fecal	LC-MS	(39)
	(II) Environment	Heat stress	Investigate the effects of chronic heat stress on serum metabolome in finishing pigs reared in monitored facilities for area, luminance, light and feeding *ad libitum*.	Pig	Serum	GC-MS	(40)
				Understanding gender specific heat stress-mediated metabolic changes and investigating metabolites potential biomarkers.	Pig	Saliva	^1^H NMR	(41)
		Preslaughter stress	Understand molecular mechanism in response to stress occurring during preslaughter period.	Chick	Muscle	LC-MS	(42)
(III) Health		Tumor	Identify metabolic biomarkers evidence of tumor presence and disease characterization with metabolic networks.	Dab	Liver	^1^H NMR	(43)
			Clinical mastitis	Discover serum metabolites and lipids that precede clinical mastitis during the imminent period in dairy cows.	Cow	Serum	LC-MS	(44)
			Periparturient period	Assess individual metabolic patterns at different stages of the periparturient period and following the changes of these patterns during both pregnancy and lactation period.	Cow	Serum	LC-MS	(45)
		Vaccine	Identify metabolomic markers of the primary and secondary immune response to administration of an intranasal vaccine.	Calves	Plasma	LC-MS	(46)
	(I) Nutrition, (II) environment, (IV) behavior	Feed, high temperature space, gender	Evaluate the potential of volatile organic compound and metabolites in animals at 12th day from birth to identify biomarkers evidence of a negative postnatal experience. Animals were deprived of feed and water and moved in bad conditions both in terms of movement and temperature.	Chick	Fecal	LC-MS GC-MS	(47)
		(II) Environment, (IV) behavior, (V) mental state	Toys and socialization	Determine the combined effects of early socialization (opened pens) and neonatal enriched environment (hearty chew dog toys, squid-shaped toys and natural ropes) during lactation (critical life phase) on the metabolome of piglets fed *ad libitum*.	Pig	Serum	^1^H NMR	(48)
			(I) Nutrition, (III) health	Diet disease	Identify compounds and metabolic perturbation involved in periparturient etiology and unravel the effect of diet on health disease.	Cow	Rumen fluid	^1^H NMR GC-MS	(49)
				Metabolic disorder	Changes in metabolomic *status* of animal during hyperketonemia.	Cow	Serum	^1^H NMR	(50)
			Study on changes of metabolites due to the development of hyperketonemia to understand functional mechanism of ketosis.	Sheep	Serum	^1^H NMR	(51)
		Analysis of lipid fraction to highlight metabolic changes during the foaling period (pre- and post-).	Donkey	Plasma	GC-MS	(52)

For each Domain, focus, purpose of analysis, animal species, type of sample, adopted analytical platform (AP), and reference number (References) have been highlighted.

AA, amino acids; FA, fatty acids; GC-MS, gas-chromatography-mass spectrometry; LC-MS, liquid chromatography-mass spectrometry; NMR, nuclear magnetic resonance; RH, relative humidity.

### 3.1. Nutrition (Domain I)

It is widely accepted by scientists that diet influences the animal as a whole entity, affecting nutrient assimilation, disease resistance, and behavior. The scientific interest in nutritional aspects in relation to AW is particularly relevant for exploring the impact of diet on AW, as well as many other features such as meat quality and sustainability in case of food producing animals. Metabolomics has been used to characterize the effects of both a deficiency or a supplementation of different nutrients in order to compare and explore the metabolic effects on opposite levels, also distinguishing all those variable factors such as age, gender, and lifestyle from diet (53). Overall, metabolomics approach proved the effects of nutritional intervention, but at the same time offers the advantage of shedding light on metabolic perturbations related to the general context in which the animal is located, allowing to acquire a more holistic view of the animal's *status*.

An interesting example showing the involvement of different Domains is the observational case-control study carried out by Carrillo et al. (35) focusing on two feeding systems. In particular, these authors applied—omics techniques, metabolomic and transcriptomic, to compare grain-fed and grass-fed beef. A clear difference between the two feed regimens was shown by changes in metabolites levels from blood and muscle tissue, mainly attributable to glucose metabolism and lipid oxidation. Also, grass-fed animals reared in their natural habits showed lower levels of circulating cortisol than the grain-fed group reared in limited spaces and fed *ad libitum*, source of competition among animals; the authors attributed this finding to the less stressful condition experienced by grass-fed animals, free to move and express their behavior (35). In this view, the integrated view of the Five Domains model for AW assessment is supported. Therefore, despite the high energy diet ensured to grain-fed animals, the environmental condition and agonistic behavior played an important role in defining the overall welfare condition, confirming the need to take into consideration more than one Domain and balance them to correctly estimate the contribute of the experiences played by nutrition (I), environment (II), and behavior (IV) Domains.

Feed supplementation with bioactive compounds of both water- and land-species animals may have an impact on AW. Metabolomics was employed by Wagner et al. (34) to investigate the metabolic profile of liver and white muscle of Atlantic salmon (*Salmo salar*) fed with vegetable oils-based diets enriched with sesamin, a lipid modulator able to convert short 18-carbon fatty acids (FA) to long chain polyunsaturated FA, to ensure their content in farmed fish tissues. Most perturbed metabolisms for liver were amino acids (AA), carbohydrate, and lipid, while for the muscle lactate, creatine/phosphocreatine, and nucleosides. According to their findings, the up-regulation of a metabolites marker of xenobiotic exposure was observed in the fish fed with high level of sesamin, since fish recognized sesamin as xenobiotic compound; moreover, high lactate levels measured in the treated salmons, were indicative of response to a wide variety of stress. Furthermore, salmon fed with high sesamin levels showed lower final body weight, especially relevant for producers (34). Turning to land animals, the effect of dietary arginine supplementation on the metabolome of growing pigs was evaluated by He et al. (33) exploiting ^1^H Nuclear Magnetic Resonance (NMR)-based metabolomics. According to their findings, L-arginine supplementation greatly affected serum concentrations of nitrogenous and lipid signaling molecules and intestinal bacterial metabolites, also suggesting a great potential for the enhancement of protein synthesis in skeletal muscle and the modulation of the gut microbiota (33). These results prompted the same authors to investigate the dietary intervention on weaning piglets to explore how arginine may relieve early life stress (54). Weaning is a piglets stressful condition that deals with (i) psychological stress, early separation from mother; (ii) environmental stress, moving to new facilities; (iii) health stress, gastrointestinal dysfunction and metabolic disorders; (iv) nutritional stress, dietary change, and (v) piglets sometimes behave more aggressively, as observed in the common increase in fighting in the new pens (31, 54, 55). He et al. observed that the arginine supplementation was more effective for weaned piglets than the growing ones in terms of growth performance, but less successful to restore the most perturbed ecology of the gut microbiota (54).

Nutritionally regulated biomarkers capable of assessing the AW *status* have been identified in farmed animals in malnutrition conditions. Nutritional deficiency is a negative experience within the first Domain explored by several authors by metabolomics. Gil-Solsona et al. (36) used gilthead sea bream (*Sparus aurata*) to compare metabolic fingerprinting of serum from fed and 10-days fasted fish by an untargeted metabolomics approach. The main perturbed biological processes were seven: FA oxidation, AA catabolism, lipolysis, gluconeogenesis, Maister's cycle, FA/phospholipid metabolism, and biotin metabolism. Higher circulating levels of fatty acyl carnitines, known as carrier of FA, were found in fasted fish suggesting that organism mobilizes all energy stores to cope with the stress condition. Moreover, elevated levels of urea cycle metabolites confirmed that both FA metabolism and AA catabolism are highly involved in negative energy balance (NEB) assuming a key role in the specific response to the condition of feed deprivation (36). On the other hand, this study highlighted the possibility to identify biomarkers concerning fish general welfare *status* since some identified metabolites were relevant for both malnutrition and pollutants toxicity (34, 36). In the context of feed deprivation of fish, Kullgren et al. (37) have obtained a general picture of which metabolites are up- or down-regulated during feed deprivation in salmonids considering juvenile rainbow trout (*Oncorhynchus mykiss*) vs. a prolonged stress condition related to 28-day fasting period. Both polar and non-polar extracts from muscle, liver, and plasma were investigated by ^1^H NMR spectroscopy analysis. A remarkable discrimination between two groups was observed, and the fasting-induced changes detected in tissues and plasma contributed to elucidate the effect of nutritional long-term stress on metabolic response. Higher levels of muscle phosphocreatine detected in fasted fish is considered the mechanism that allows fish to keep readily available energy for burst swimming and important behavior responses -such as foraging activities and predator avoidance—during fasting, contributing not only to the survival, but also to the general fish welfare *status* (37).

### 3.2. Environment (Domain II)

Environmental Domain opens the window to a wide range of variables to be considered. For food producing animals, depending on the outdoor or indoor type of rearing, animals may be exposed to uncontrollable extreme weather conditions or, on the contrary in confined spaces to an overcrowding condition; all these discomforts may cause severe physiological stress and debilitating state, even pathologies, for animals (9). One of the most widely explored environmental stressor is heat stress that, in energy terms, refers to a particular NEB since the amount of heat produced by the animal may exceed the capacity to dissipate the heat in environment, creating an imbalance. This state can be defined by external variables, e.g., air temperature and humidity, and internal variables specific of the animal, e.g., species (56). Temperament, genetics and the early life experience (i.e., maternal contact during development stage) play a pivotal role in determining how animals, both during childhood and adulthood, experience the environment. In fact, the neural function of animals is strictly related to epigenetic factor: new sensory experiences may alter genes expression in discrete regions thanks to the plasticity of central nervous system. In this sense, a key physiological role is played by hippocampus. Therefore, animals may experience the same environment in different ways (14, 57–59). A common response of animals to heat stress is the decreasing in feed intake to silence all those processes generating heat—such as digestion—with the aim to survive (60). In case of investigations dealing with thermal stress, environment is the main Domain of interest, but the repercussion on other Domains, i.e., nutrition, is tangible. This involvement needs to be taken into consideration for an integrated assessment of AW enabling to develop adequate management strategies able to minimize or reduce the negative consequences of heat stress.

In the swine sector, Cui et al. (40) investigated metabolome changes in serum of finishing pigs exposed for 3 weeks to 30°C, compared to a thermal neutral condition of 22°C, aiming to discover potential biomarkers related to heat stress. Five metabolic pathways were considered relevant for the description of chronic heat stress exposure, as follows: carbohydrates, AA, amines metabolism and gut microbiome-derived metabolism. Pigs showed decreasing in feed intake and glucose levels leading to NEB, partly attributable to the decrease in concentration of 6-phosphogluconic acid; as a short-term compensatory mechanism, the finishing pigs used glucose precursors, protein, and AA, through the deamination process and the gluconeogenesis, to overcome the lowering levels of glucose in the serum. A role in NEB adaptation has been shown by ketone bodies and non-esterified FA, whose levels were elevated in the stressed group. These findings confirmed that heat stress condition urges the mobilization of all energy stores to cope with the stress; however, particular attention has been paid to the higher levels of gut-microbiome metabolites observed in pigs reared at high temperature whose induced an increased permeability of intestine conducting to systemic inflammation. This last remark is fundamental in view of a general AW assessment because the symptoms related to the disease (health, Domain III) are the outcome of the activation of a series of biological pathways triggered by multiple stress originating from different Domains (environment, Domain II; nutrition, Domain I). Metabolomics also revealed alteration of phospholipid and FA composition during heat stress (40). The involvement of lipid metabolism in thermal stress response was observed by Qu and Ajuwon (38) in adipocytes differentiated in culture and mesenteric adipose tissue of pigs exposed to extreme temperature conditions. *In vitro* and *in vivo* settings showed distinct metabolite profile, reflecting the *in vivo* complexity of heat stress adaptation. Moreover, a gender effect was observed by the Principal Component Analysis (PCA) of mesenteric fat that showed a better separation of metabolites in the boars than gilts, suggesting a further variable (gender) of the adaptive response to heat stress in pigs (38). Untargeted metabolomics analysis was performed by Zhou et al. (39) to explore the combined effects of air temperature and relative humidity on metabolic pathways in broilers. Fecal samples of broilers assigned to three different treatments—reared, respectively, at 35, 60, or 85% relative humidity (RH) with a gradually increased temperatures to 32°C, over the course of 15 days—were investigated. Based on the results, the authors made evidence that RH affects several metabolic pathways (in particular glucose, adenosine triphosphate–binding cassette transporter metabolisms, and the aminoacyl-tRNA anabolic pathway, metabolic pathway of taurine and hypotaurine) associated to heat dissipation and growth decline, confirming that environmental stress may induce perturbations on different Domains contributing to the definition of the overall wellbeing of the animal (39).

### 3.3. Health (Domain III)

Animal health is defined as the ability of the animal to adapt to the pathology (31). It is widely accepted that health is a key part of welfare representing the condition of existence for the AW definition. Sources of threat to health domain can be metabolic disorder (i.e., ketosis for dairy cows and ewes), mycotoxin exposure (i.e., fumonisin causing leukoencephalomacia in horses and pulmonary edema in pigs), skin lesions (i.e., aggression in pens) and other unpleasant events (i.e., tail biting with evidence of chewing and possible infection) (43, 61–63). Metabolomics has been used as a tool to explore the health (Domain III) in relation with other aspects, in particular environment (Domain II) and nutrition (Domain I). As an example, Southam et al. (64) applied the NMR-based metabolomic profiling and correlation networks alongside metabolite fingerprinting, to explore the metabolic network involved in hepatic tumors of flatfish, species used for environmental monitoring as they live close the marine floor where toxicants and carcinogen agents can accumulate in the sediment. Therefore, the dab (*Limanda limanda*), a disease sentinel used in European marine monitoring programs, was chosen for this study. Metabolic differences between two phenotypes (health vs. diseased) were small, effect probably masked by the high intragroup variability; for this reason, after histological evaluation of neoplastic lesions, the comparison between metabolome of healthy and tumor tissues sampled from the same liver was considered. Changes in metabolites abundance were clearly demonstrated, perhaps accountable to tumor development stage. Also, an increased anaerobic respiration—supported by the higher levels of lactate, succinate, and acetate—and the depletion of choline were evidenced in tumor tissue, leading the authors to hypothesize that disruption of the choline oxidation pathway may be the key metabolic change in flatfish liver tumor enabling DNA hypomethylation and oncogene activation (64).

As mentioned above, feed represents not only an important factor defining AW *in toto* but also a source of threat for animal health, in the case of mycotoxin-contaminated feed. In that context metabolomics proved to be a powerful technique to investigate the relationship between health and nutrition Domains. In particular, Wu et al. (65) applied metabolomics to study the relationship among metabolic changes mycotoxin-induced, mycotoxin contaminated-feed (treat), and feed supplementation. In detail, both the toxic effects of deoxynivalenol (DON) on pigs and the effects of supplemental glutamic acid on DON-induced toxic damage in piglets were investigated throughout an NMR-based metabolomic approach. The findings indicated that glutamic acid may be a useful nutritional supplement for regulating DON-induced injury, being able to decrease oxidative stress, promote intestinal epithelial cell proliferation, and regulate energy, lipid, and AA metabolism disorders induced by the dietary exposure of trichothecene mycotoxin of pigs (65).

### 3.4. Behavior (Domain IV) and mental state (Domain V)

Currently, very limited scientific evidence is available concerning metabolomics studies addressed to animals' emotional experiences within the welfare context. Despite this, the theoretical framework supports the assumption that the welfare of an individual is the totality of its efforts to live positive experiences in each of the Five Domains, suggesting that both mental state and behavior are very impactful topic to explore (66). A strong correlation between behavior and mental state may be assumed because animals manifest feelings through specific behaviors (67) following stimuli such as social interaction (e.g., separation or socialization), mental stimulation, stress (e.g., weaning for piglets) as well as nutrition, environment, and health (68). There is a wide consensus that cognitive development may arise from environmental inputs such as the interaction with new material or sensory stimuli. In the pig sector, behavioral development and stress adaptation are positively affected by an enriched environment during the early life of piglets and social interaction between litters during lactation (69). The mechanisms underlying the decrease in agonistic behavior and reduced stress response in relation to the combined early socialization and environmental enrichment were successfully investigated by a recent study of Saladrigas-García et al. (48) throughout an untargeted metabolomics approach. The authors performed the study comparing pigs reared with enrichment objects (i.e., hearty chew dog toys, squid-shaped toys, and natural ropes per pen) vs. control group reared with standard management system. As a proof of differences, molecules related to energy metabolism (triglycerides, FA, very low- and low-density lipoproteins, and creatine) were significantly lower in the piglets of enriched environment probably due to the fact that pigs are engaged in playing with enriched material by reducing pen interaction, thus minimizing energy requiring. As a consequence, a reduction of aggression in these animals was observed suggesting that physically and socially enriched environment in early life can modify animal response after weaning, probably by means of diminishing social stress response. In fact, control group showed an increase in body lesions supported by the higher levels of stress markers, such as salivary cortisol and chromogranin (48). However, to the best of the author's knowledge, the application of metabolomics approach to Domains IV and V of the Five Domains model is lacking, and further work on these issues is encouraged. For example, an abnormal behavior, which is detrimental to both welfare and economy and for which metabolomics might provide adequate insights, is tail biting in pigs, for which a pig bites the tail of pen-mates (70). This behavioral tendency is the manifestation of a set of several sub-optimal living conditions regarding nutrition, environment, health, and other triggering cause (71). So far, only targeted approaches have been employed to explore the issue. Assuming that the tail biters may suffer from the inability to absorb nutrients, Palander et al. (72) focused their study on the determination of blood minerals and AA and gut cell wall structure. According to their findings, no signs of nutritional deficiency in tail biters on the basis of their intestinal morphology or blood metabolites were observed. On the other hand, data suggested differences in relation to the tail-biting environment and being bitten. Free access feeding (nutrition, Domain I) with restricted feeding space (environment, Domain II), compared with feeding twice a day with unrestricted feeding space, was associated with an overall reduction in AA levels in plasma and deepened crypts in the jejunum. In this context, metabolomics could be exploited to identify metabolic changes enabling to recognize early signs leading to tail-biting behavior and formulate appropriate remedial strategies (72).

## 4. Metabolome analysis

The study of the metabolome is considerably complicated being highly susceptible to external variations. Depending on the aim to pursue, metabolomics research may be performed following a targeted or untargeted strategy, mainly different in terms of metabolites coverage, being the first pointed to a selective recovery of groups of metabolites, whereas the second one aimed at maximizing the number of metabolites. Targeted strategy is a hypothesis testing approach and generally, throughout known chemical compounds used as internal standards, provides the quantification of targeted molecules (73–75). The untargeted strategy is aimed at characterizing the whole metabolome and designed for the identification of as many metabolites as possible, up to thousands of molecules, both known and unknown (or novel) providing information about changes in metabolites (27, 75). Combining targeted and untargeted strategies can be considered a potential choice for metabolomics studies since this integration may help to pinpoint and quantify metabolites (53, 76, 77). The biological understanding from metabolomics experiments relies on a good experimental design, after the formulation of a proper biological question (78).

Most of AW metabolomics studies are observational case–control studies and focused to grab the molecular response generated from the exposure to different factors belonging to individual Domains. Generally, studies consist of two or more groups of animals, respectively, identified as *control* and *treatment/s* (35, 39), but longitudinal studies are also considered to investigate AW aspects mainly related to Health (IV) and Nutrition (I) Domains (49, 79). A critical aspect concerning the study design is being able to consider all the conditions affecting AW simultaneously, because also ensuring the best living conditions, the interaction between the animal's previous experiences and temperament may completely reverse the situation. Animals' feelings eliciting certain behaviors are still a challenge that could find answer by the metabolome studies. Comprehensive planning of complex experiments may take advantage from the Design of Experiments (DoE), a statistical methodology to plan experiments efficiently (80), although no evidence is available for metabolomics studies.

Sample size is a pivotal issue to enable detection of statistical significance, but there are not standard methods for its estimation in metabolomics. And so, as often happens, ethical and economical restrictions mainly determine the number of samples (e.g., animals to be sacrifice) for a study (81). Classical univariate sample size determination is usually not straight forward for highly dimensional and multi-correlated metabolomics data (78). The minimum sample size may be estimated by a power analysis method described for high-dimensional metabolomics data (82). Alternatively, an analysis-based approach by publicly available software package for selecting the optimal sample size has been proposed by some authors (83).

The choice of sample type, biofluids and/or tissues, relies on the objective of the investigation, although in longitudinal studies biofluids are the preferred choice for repeated measurements. In terms of feasibility of sampling, biofluids such as urine, stool, and saliva can be collected non-invasively, whereas blood and tissues' collection are much more complicated and may be arduous, therefore qualified personnel is required (84). Changes in metabolome of saliva, serum, and adipose tissue were used to study the effect of heat stress in pigs (38, 40, 41). Both biofluids and tissues were considered to gain a global view on metabolic processes and elucidate the effects of feed deprivation in salmonid fish (37) and in an integrated multi-omics study aimed at ascertaining the effects of the finishing forage on metabolic pathways related to AW and meat quality in bovine (35).

### 4.1. Metabolomics workflow

Metabolomics is one of the -omics techniques requiring wide and robust analytical strategies. By and large, scientists agree that metabolomics workflow consist of sample preparation, sample analysis, data processing, data analysis, and biological interpretation (85, 86).

#### 4.1.1. Sample preparation

The ideal sample preparation for metabolomics analysis should guarantee high recovery of metabolites and be as rapid and simple as possible—to minimize degradation reactions and exogenous interferences—as well as reproducible. As a matter of fact, even minor variations in the centrifugation of plasma can affect the recovery of some metabolites, which can be degraded or created after the sample's extraction procedure (87, 88). The broad number of metabolites different for chemical structure, reactivity, and concentration make the simultaneous extraction of all molecules challenging and no single method can cover the entire metabolome.

Sample preparation heavily depends on the approach, targeted or untargeted. In the first case, conditions are optimized according to the physicochemical properties of metabolites, in particular polarity, to maximize their recovery. In the second case, sample treatment aims for increased coverage of metabolites, often leading to suboptimal recoveries for specific compounds: this justifies the introduction of lipidomics to refine sample preparation enhancing the recovery and study of lipids (73, 76). For tissues, the sample representativeness may be an issue because organs may be described and respond regio-specifically; for example, the liver presents five different topographic lobes characterized by different levels of some enzyme systems expression, as well as the kidney consists of medulla, cortex, and many cell types with different structures and roles (89). Generally, 1–100 mg of tissue and 10–100 μl of biofluids are sufficient (34, 37, 41, 90), although the amount relies on the type of the analytical platform used for the analysis.

Challenges, pitfalls, and best practices to follow in sample preparation for metabolomic studies of biofluids, tissues, and mammalian cells have been extensively described in literature (91, 92). However, some trends and issues in animal sample preparation are worth mentioning: such as for preliminary handling step, quenching may be necessary to switch off the enzyme activities of samples by extreme treatments based upon pH or temperature changes (92). Dehydration of the sample may also be used for this aim, although water removal may cause metabolites' adsorption on cell walls and membranes leading to untrue results (20). Frozen tissue should be thawed on ice to reduce changes in sample structure (89).

Literature often shows the use of biphasic extraction protocols to recovery simultaneously both polar and non-polar molecule classes. Among them, the Folch's and Bligh-Dyer methods, and their modifications, are those most widely employed (93, 94). Both are two-step protocols based on the use of a mixture methanol/chloroform in different ratios and water. For environment and operator safety purposes, dichloromethane and methyl *tert*-butyl ether have been proposed as solvent alternatives to chloroform. The use of water helps to split the system in a polar upper layer and a non-polar lower one (95).

#### 4.1.2. Sample analysis

Actually, none of the analytical platforms can detect the great heterogeneity of the metabolites. Nevertheless, NMR spectroscopy and Mass Spectrometry (MS) are the two major analytical platforms pillars adopted in metabolomics.

MS-based analysis provides a spectrum that sums up measurements of mass-to-charge ratio (*m*/*z*) and the relative abundance of ionized molecules (96). Experiments MS-based may consider several options for separation and detection (53). Both gas- and liquid-phase separation are employed, exploiting physico-chemical properties (i.e., volatility of metabolites, interaction with phase in a column) of compounds (28, 97). Application of capillary electrophoresis-MS are limited, despite this technique is particularly suitable for analysis of volume-limited biological samples (98, 99). Various types of detectors have been developed to improve the performance of analysis in terms of enhanced sensitivity and selectivity such as quadrupole, quadrupole ion trap, and time-of-flight (ToF) are the mass analyzers commonly used (84). For targeted analysis purposes, triple quadrupole is the favorite choice because suitable for quantitative analysis by low-resolution MS. On the contrary, for untargeted applications, also known as full scan approach, high resolution MS (HRMS) may be preferred (92). Among the emerging MS techniques no requiring prior sample preparation, MS imaging is very promising because it has the advantage of visualizing the distribution of metabolites in tissue or cells making this approach less time consuming and solvents employing (100, 101).

NMR spectroscopy proved to be a powerful tool for structural and quantitative information but also to identify unknown metabolites (102, 103). Considerable progress has been made in NMR-based metabolomics considering that it is highly reproducible and allows the identification of compounds with identical masses; on the other hand, it provides the detection of most abundant metabolites being less sensitive than the hyphenated methods (97, 103). Literature offers many examples of NMR-based metabolomics applications showing that the most commonly investigated nucleus is the proton ^1^H, having a natural abundance of 99.98%, but also other nuclei such as ^13^C, ^15^N, and ^31^P can be investigated (103, 104). A critical aspect concerning the employment of NMR spectroscopy in metabolomics is that biological samples are complex matrixes characterized by the presence of proteins, lipoproteins, and water, whose signals in the spectrum may hide those metabolites at lower concentration. To overcome these issues, several pulse sequences have been developed to improve specific NMR signals. For water suppression, NOESY pulse sequence is the most popular applied for the acquisition of metabolomics profile (105), specifically, the ^1^H NMR spectra of the polar extracts of both biofluids and tissues are usually recorded (34, 106). In the case of darkening-signals macromolecules, the Carr-Purcell-Meiboom-Gill (CPGM) pulse sequence may be employed to overcome the problem related to low concentration metabolites (105). For example, the authors Le Roy et al. employed CPGM sequence for the acquisition of the ^1^H NMR spectra of chicken's plasma and egg white to limit the contribution of albumin and ovoalbumin signals, respectively (106). Further, NMR spectrum may be severely affected by pH, osmolality, and concentrations of some ions creating a variable condition in ppm chemical shifts of proton signals (92, 96). In this regard, it is recurrent the use of a buffer solution with pH = 7.0/7.4 during sample preparation to control ion concentration (34, 37, 106).

#### 4.1.3. Data processing

NMR and MS provide a considerable amount of data, of the order of thousands signals, which form the data sets of the putative metabolites. Initially, raw data sets contain undesirable information such as noise (107). This unnecessary information might force some trends at a later stage and, at first, alleged interferences must be removed by adequate data pre-treatments (102). Further data processing consists in setting the raw data into a format that can be used for the subsequent data analysis. To combine data from different samples, the alignment of signals is needed because often shifts in ppm and in retention time are observed in NMR and MS, respectively (102, 108). To this aim, an internal standard may be added during sample preparation and used as a reference signal. Unwanted data variation related inevitably to both experimental and biological sources such as sample preparation, analysis of multiple batches, inter-instrument and inter-laboratory variation, constitution of the biological samples, may be removed by normalization. This step minimizes the risk of identifying false biomarkers, missing out true biomarkers, artificial classification or clustering of the samples or metabolites (109, 110). Scaling is a step of data processing that places each metabolite in a comparable scale because metabolite levels may range over many orders of magnitude (111). Autoscaling and Pareto scaling are the most commonly scaling methods used in metabolomics (97, 102).

#### 4.1.4. Data analysis

Metabolomics data require reliable statistical methods, defined as chemometric tools, to efficiently extract the maximum useful information. For this purpose, the multivariate data analysis (MVDA) is generally employed to manage large and complex data sets, for data visualization and exploration, and the identification of patterns (112, 113). For explorative aim, PCA is often the first step applied to detect the presence of natural grouping trend or outliers by displaying with scores plot the relation among the observations or samples in the model plane. Hierarchical cluster analysis (HCA) is also used for preliminary evaluation of information contents in the data to study any differences or similarities, with a separative or agglomerative approaches, characterizing the investigated samples. The relationship between the samples is graphically summarized as a tree plot building the dendrogram, where the contribution of each variable is shown by an heatmap (114). In the study of Carrillo et al. PCA and HCA revealed a perfect separation between grass- and grain-fed Angus steers based on the metabolite profiling from muscle tissue and blood, that may be indicative of the divergences in global metabolism (35). According to Qu and Ajuwon metabolomics of heat stress response in pig adipose tissue revealed alteration of lipids during heat stress, and PCA provided a separation of metabolites by temperature (38).

Unlike PCA and HCA that do not take into account the information about the class membership (i.e., control vs. positive, exposed vs. non-exposed, feed supplementation vs. normal diet), Partial Least Squares-Discriminant Analysis (PLS-DA) and Orthogonal Partial Least Squares- Discriminant Analysis (OPLS-DA) are discriminating analysis conducted for improving separation between groups by using class information (97). In this framework, the contribution of metabolites in the discrimination is expressed by the Variable Importance in Projection (VIP) score, where the greater the VIP score, generally with a score value higher than unit, the greater the contribution to the separation between sample groups. For example, in a metabolomics study aimed at identifying biomarkers of malnutrition in farmed fish, using gilthead sea bream (*Sparus aurata*) as a model, PLS-DA clearly discriminated the fasted individuals from those of the fed group; moreover, by OPLS-DA around 850 features of the data set were highlighted as discriminatory between fed and fasted fish (36). In the study performed by Zhou et al. (39) focused on metabolic profiling of chicken stools within environmental stress characterization, PLS-DA plots showed a clear separation between groups of broiler chickens reared at different relative humidity and temperatures. Moreover, 36 metabolites with VIP score >1 were identified and further investigated as potential biomarkers related to hot humid or dry stress (39).

#### 4.1.5. Biological interpretation

Statistical significance does not mean biological significance, and biological interpretation should not be derived on the change of biomolecules selected by statistical significance. Biological interpretation is the most challenging step and the real bottleneck of metabolomics workflow. Metabolism is a complex and dynamic network where known metabolites are connected by enzymatic and non-enzymatic reactions or structural similarities. Once a list of metabolites possible candidate biomarkers has been ranked by data analysis, bioinformatics is needed to support the metabolomics interpretation. Pathway analysis, metabolite set enrichment analysis, and metabolite correlation networks are the tools most frequently applied to gain biological insights (112). For example, Zhou et al. (39) applied pathways analysis to identify pathways affected by hot-humid or dry climate in broiler chickens reared in different conditions of relative humidity. In particular, in this study the relevant metabolites were subjected to Metaboanalyst, a free online tool based on the KEGG metabolic pathways database; thirteen metabolic pathways were detected as enriched metabolic pathways for the hot-humid or dry stress, suggesting that those environmental conditions can affect all those metabolites involved in fat synthesis and associated with skin vasodilation and blood flow (39). In the NMR-based metabolomics investigation of Southam et al. (64) addressed to hepatic tumors of flatfish, metabolic correlation networks analysis revealed increased anaerobic metabolism and reduced choline metabolism in diseased tissue compared to healthy phenotype. Moreover, significant negative correlations were observed between alanine-acetate and between proline-acetate in diseased tissues only, suggesting that alanine and proline are utilized as alternative energy sources in flatfish liver tumor tissue (64).

## 5. Metabolomics and animal welfare: A step forward

Metabolomics proved its effectiveness in clarifying biological cause-effects mechanisms of living organisms, confirming that the phenotypic understanding can take place on molecular scale by investigating metabolite identity and abundance. Focusing on a set of metabolites may provide significant clarification on cellular regulatory processes involved in the latest response of the biological systems to genetic and environmental changes.

Therefore, this approach can metabolically answer to biological questions concerning AW issues even when applied to individual Domains, hence with a limited perspective and covering confined areas of welfare. As a matter of fact, none of the studies listed in Table 1 answered the key question *Did the animal live in a good welfare status?* In fact, metabolomics applications show how changes in metabolome can be induced by different factors such as diet, environmental stress, health, and mental state, which are the same factors declared from the Five Domains model. Thus, successful results have been achieved considering the Five Domains individually; in other words, all described examples show that the condition under investigation is targeted toward a well-defined condition i.e., nutrition state. Although satisfactory, it may be a limiting strategy supported by the assumption that AW cannot be described on the basis of individual Domains considering, for example that a stressful condition can have consequences embracing all blocks of the Five Domains model, as in the case of mycotoxin-contaminated feed (65).

Overall, the Five Domains model has been poorly explored in its completeness with metabolomics. Thus, promoting linking and combining the theoretical and scientific frameworks—the Five Domains model and metabolomics—could be the turning point to make available objective tools enabling to fill the gaps in AW assessment. This cue is strongly supported by the holistic nature of metabolomics approach which provides a dynamic view of biological systems integrating the flow of information from DNA, RNA, and proteins, ensuring a comprehensive overview of living systems. In fact, in the hierarchy of omics methodologies that reflect the molecular expressions of living systems, metabolomics is the most sensitive to variations resulting from a complex interaction between genotype, lifestyle, nutrition, drug therapy, and environmental exposure (20, 115); in this way it may capture and integrate all information relevant for the molecular definition of AW phenotypic outcome. Therefore, metabolomics might be exploited at the best for the evaluation of the wellbeing *status* of the animal as a whole entity, and unravel biomarkers intended as a characteristic that is objectively measurable reflecting a given condition (29, 30).

In the opinion of the authors, there are some challenges to face for the successful application of metabolomics in the setting of AW mainly related to the following two questions.

### 5.1. Might be the metabolomics approach a tool supporting the current AW assessment models?

**Figure 2:**
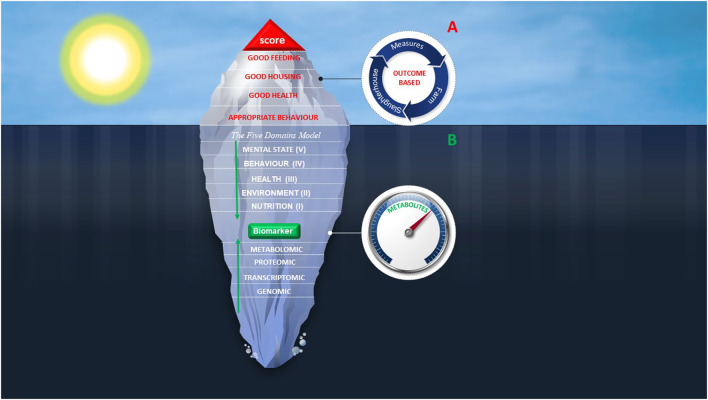
Iceberg represents the proposal of a model for the future AW assessment. Combining scoring systems **(A)** with metabolites identification and quantification **(B)** may greatly improve the understanding of welfare condition and assessment. **(A)** Tip of the iceberg represents the current AW assessment protocols focused on management-based requirements and animal-based measures. Red items are welfare criteria on which are based measures on farm or at the slaughterhouse. These protocols provide a score indicating if welfare criteria are fulfilled. **(B)** Underwater part of the iceberg represents a new path for AW assessment that includes an integrated view of the Five Domains model. All Domains can affect the phenotypic outcome of living organism which can be explored through the metabolomics approach. The Five Domains model meets untargeted metabolomics in the molecules measuring.

Currently, AW assessment models are outcome-based systems obtained by adopting scoring systems as an evaluation metric. These models focus on the compliance with functional and management requirements as well as animal-based measures, all of them considered iceberg indicators of welfare (Figure 2A). In the opinion of the authors, coupling the Five Domains model with metabolomics might be the solution to provide the underwater part of the iceberg enabling for a whole picture of AW. By providing insights into metabolome, metabolomics could make available novel biomarkers spanning all Five Domains and that might be useful items for a comprehensive assessment of AW *status* (Figure 2B). Moreover, in this framework, the corrective actions will be targeted toward solving the problems due to the fact that metabolites are considered the “canaries” of genome and this nature as sensor might also be successfully employed as a preventive screening measure (116).

### 5.2. Which experimental workflow might be more powerful to give novel metabolic insights related to AW?

A well-conceived experimental design should be accomplished to gain a molecular snapshot as closer as possible to reality related to the condition of animal wellbeing. We have good reasons to think that further progress in analytical and computational technology will give even more performing tools able to increase metabolite coverage and biological interpretation, still the bottleneck of metabolomics workflow. It may be reasonable believe that carefully defining the study design might help in understanding the biological meaning of AW by remarking the idea that a condition in which animals live might be biologically demonstrable. In view of integrating metabolomics and Five Domains model, efforts should be addressed to include in the experimental studies animal behavior and mental state that, as far as the authors know, are the Domains least explored by metabolomics.

## 6. Concluding remarks

The multidisciplinary nature of AW, well-represented by the different items according to the Five Domains model, makes the development of tools for the objective assessment of the AW *status* challenging. Based on the literature, most of the metabolomics studies considering AW have approached the topic focusing on the study of one single Domain, with a limited perspective. Nevertheless, metabolomics has shown effectiveness in the discrimination between groups of animals subject to different conditions, the evaluation of the most perturbed metabolisms, and the detection of biomarkers. As a whole, even individually considered by metabolomics approach, the investigated conditions are able to induce tangible outcomes in the metabolic profile of the animal, by allowing for the correlation of biochemical changes with phenotype.

In this regard, in the last decades metabolomics has generated a remarkable amount of data through the improvement of high-throughput technologies both as analytical platforms and data analysis strategies. However, specifically in the context of AW, metabolomics is still in its infancy, and the way to getting full coverage of all relevant metabolites is still long. Progress in metabolome coverage confidence and standardization of approaches across the entire workflow is needed for metabolomics to become a mature application to be widely adopted.

Within these limitations, efforts of the metabolomics scientific community have been made to gain meaningful biological and chemical insight from complex metabolomic data. Deciphering metabolites to be used as biomarkers of comprehensive AW *status* is a promising tool that may pave the way for new control strategies for both living animals and the food products thereof. Moreover, increased knowledge of metabolic pathways and biological interpretation will allow defining management strategies to improve the welfare in food-producing animals in livestock systems.

Looking forward, the inclusion in the experimental design of the last two Domains, behavior and mental state respectively, as well as nutrition, environment, and health, would represent a turning point for the integrated application of Five Domains model on molecular scale exploiting metabolomics. Regarding the biological interpretation, the final achievement to be reached, the combination of multi-omic data is a promising perspective but still a challenge to overcome. Further work on these issues is therefore encouraged.

## Author contributions

All authors contributed to the conceptualization, writing, reading, and approval of the final manuscript.
